# VOC emissions influence intra- and interspecific interactions among stored-product Coleoptera in paddy rice

**DOI:** 10.1038/s41598-018-20420-2

**Published:** 2018-02-01

**Authors:** Giulia Giunti, Vincenzo Palmeri, Giuseppe Massimo Algeri, Orlando Campolo

**Affiliations:** Department of Agriculture, University “Mediterranea” of Reggio Calabria, Loc. Feo di Vito, 89122 Reggio, Calabria Italy

## Abstract

Olfaction is a pivotal sense for insects and granivorous pests may exploit grain volatiles for food selection. *Tribolium confusum*, is a secondary pest of stored cereals that benefits from primary pests’ infestation, as other secondary feeders, triggering competition. This study aimed to evaluate the preferences of *T. confusum* females toward different-infested paddy rice, highlighting the impact of intra- and interspecific competition. *Tribolium confusum* showed positive chemotaxis toward rice infested by larvae of a primary pest (*Sitophilus zeamais*), but not for grain attacked by adults alone. Furthermore, kernels concurrently infested by a primary (*S. zeamais*) and a secondary pest (*T. confusum* or *Cryptolestes ferrugineus*) were evaluated in Y-tube bioassays, highlighting that both food-sources were innately attractive for *T. confusum* females. Moreover, females positively oriented toward rice infested by conspecifics, while they avoided grain infested by *C. ferrugineus*, averting an extremely competitive habitat. Behavioural responses of *T. confusum* females and volatile emissions of different-infested rice highlighted the occurrence of plant-mediated interactions among insects from the same trophic guild. Seventy volatiles were identified and significant differences among the tested food-sources were recorded, emphasizing the presence of 6 putative attractants and 6 repellents, which may be useful biocontrol tools.

## Introduction

Stored product protection is a key issue in the food production and processing contexts. Worldwide annual post-harvest losses attributable to granivorous pests have been estimated at 10% of the total cereal grain yield, determining also severe weight losses (i.e. dry matter loss)^1^. Chemical control approaches are generally preferred by food producers since they are cheapest and cost-effective^2^, although also prevention procedures^3–5^ and physical control methods^6^ are commonly employed. However, since chemical control is becoming even more strict-regulated and restrictive, researches have started to focus on the development of innovative and sustainable strategies, involving also natural enemies^7^ and plant-borne molecules^8–11^. Granivorous insects attacking stored cereals can be divided into internal and external feeders consistent with their feeding habits, which are also known as primary and secondary pests, respectively, according to typical infestation order. *Sitophilus zeamais* (Motschulsky) (Coleoptera: Curculionidae) is one of the most common and destructive insects of stored cereals worldwide. The adult weevils can attack whole grains and the females lay eggs inside the kernels, in which the larvae feed and develop^12^. Feeding activity of a primary pest is essential to boost external feeders’ survival, since these pests preferentially forage on damaged or previous infested kernels. The confused flour beetle, *Tribolium confusum* J. du Val (Coleoptera: Tenebrionidae), and the rusty grain beetle, *Cryptolestes ferrugineus* (Stephens) (Coleoptera: Cucujidae), are two economically important beetle species that typically are unable to feed on undamaged grain. Co-occurrence of *Tribolium* spp. and *Cryptolestes* spp. infestation is frequent and the magnitude of competition on the respective life-history traits has already been investigated^13^, highlighting that competitive dominance mainly depends on population density and abiotic factors^14,15^.

Olfaction is a key sense routing interspecific communication in insect communities and the role of infochemicals steering interactions among insects belonging to different trophic levels has been extensively investigated^16–18^. Nevertheless, the impact of olfactory cues shaping the relationships among insects from the same guild, competing for shared resources, is far less explored. For instance, signals left by close related species, as oviposition and host-marking pheromones, may be exploited by herbivores and natural enemies to detect competitor activity^19–21^. Plant-borne volatiles play a role in food and host location, routing insect orientation and searching behaviour. Herbivore-induced plant volatiles (HIPVs), produced by the plant in response to phytophagous feeding activity, are known to be pivotal for parasitoid host location^22,23^. Overall, HIPVs are acknowledged to affect community dynamics by influencing the performances of various members^24^. Compared to field conditions, granivorous insects attacking foodstuff rely on foraging-environments presenting simplified community structure. Although many foodstuff insects are originally polyphagous, when foraging in anthropized habitats, they can rely on few different food resources and the association between pest species is common^25,26^. Therefore, infochemicals are pivotal to detect suitable food sources^27–29^ and can improve pest searching efficiency, by shaping trophic interactions and competitive coexistence^30^. Interspecific competition, either occurring as exploitation (i.e. without behavioural interactions) or interference (i.e. through aggressive behaviours), is widespread among granivorous insects and can lead to severe consequences for the involved species^31,32^. In this scenario, the prompt recognition of unsuitable or harmful habitats is a key cue to enhance pest survival and fitness, and thus semiochemicals emitted by infested kernels could be exploited to overcome competition establishment.

Here, we focused on the olfactory responses of the external feeder *T. confusum* toward rice sources with different infestation status, to highlight the impact of intra- and interspecific interactions and the effect of primary and secondary pest infestations. Firstly, we evaluated the naïve attractiveness of intact and insect-damaged rice, testing the incidence of *S. zeamais* larvae and/or adults, as well as the co-occurrence of a secondary larval-infestation by *T. confusum* or *C. ferrugineus*. Then, the comparative preferences of *T. confusum* females for intact and *S. zeamais-*infested grains were tested in two-choice assays, in order to highlight the impact of infestation by a primary feeder on confused flour beetles’ chemotaxis. Furthermore, to assess the effect of competition among insects belonging to the same trophic guild, we evaluated the attractiveness of kernels concurrently infested by a primary (i.e. *S. zeamais*) and a secondary pest (i.e. *T. confusum or C. ferrugineus*) by comparing them to rice damaged by *S. zeamais* alone. Lastly, to determine the release of semiochemicals that can route insect orientation, and to identify Volatile Organic Compounds (VOCs) acting as attractants or repellents, the emissions of different-infested rice were sampled by Head-Space Solid Phase Micro-Extraction (HS-SPME) and analysed through Gas-chromatography/Mass spectrometry (GC/MS) techniques.

## Results

### Behavioural responses of Tribolium confusum females toward different rice-sources

**Figure 1:**
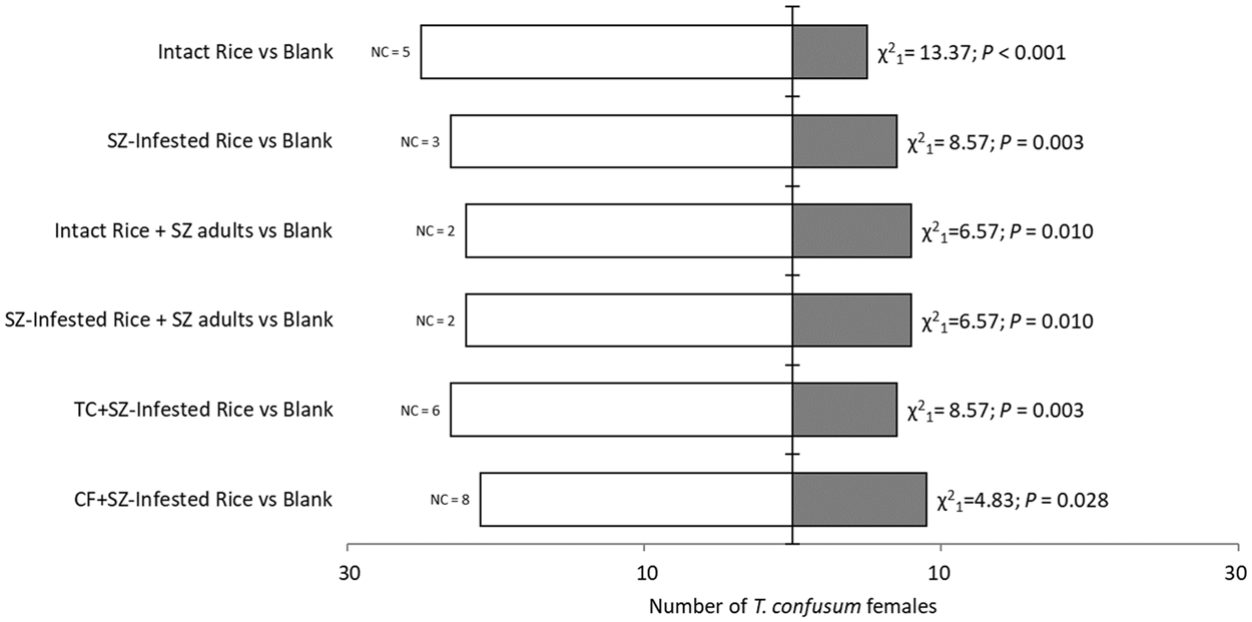
Innate attractiveness of intact and insect-damaged rice kernels toward *Tribolium confusum* females. Choice bioassays were conducted in Y-tube olfactometer comparing every odour source (5 g) vs blank. Thirty responsive beetles were tested for every comparison. For each comparison, χ^2^ and *P* value are provided. Subscripts reported for χ^2^ represent the degrees of freedom. SZ = *Sitophilus zeamais*, TC = *Tribolium confusum*, CF = *Cryptolestes ferrugineus*; NC = Number of beetle females who performed no choice between the two given cues.

**Figure 2:**
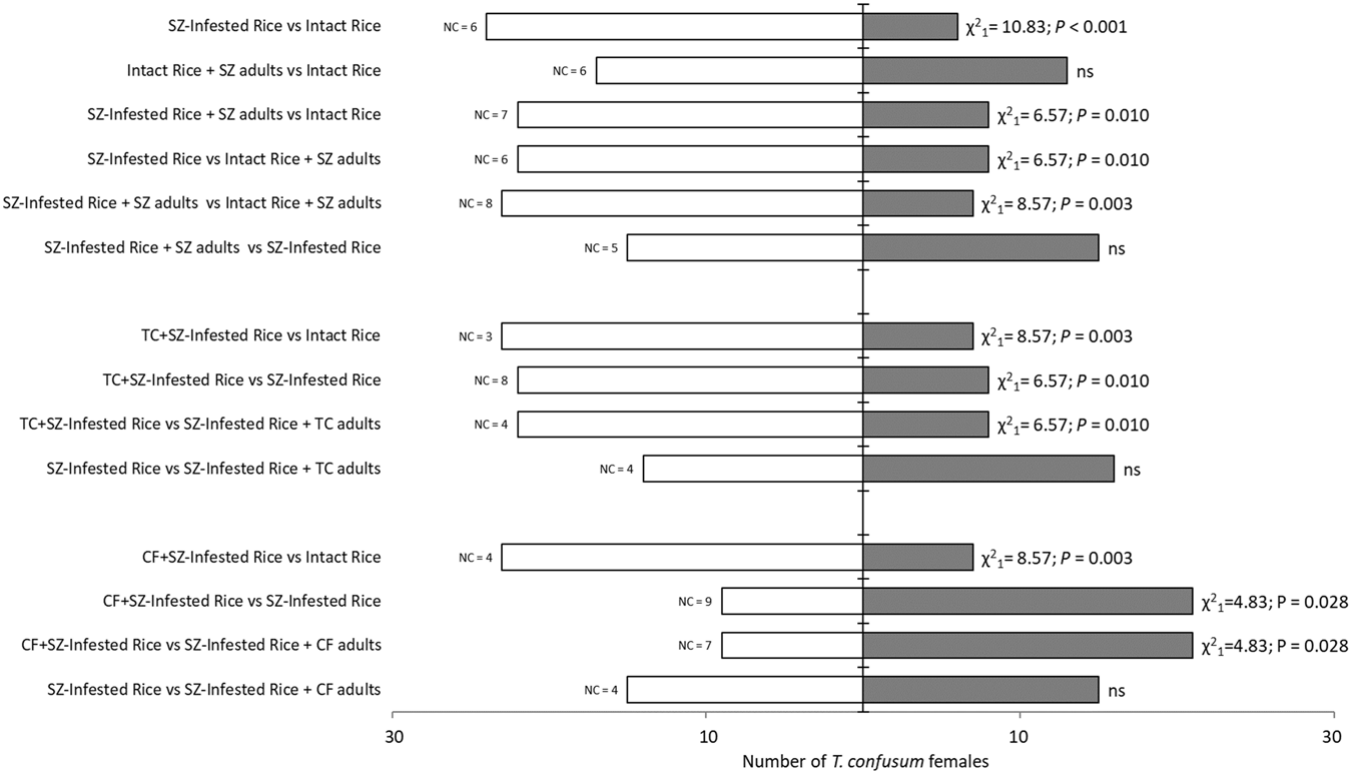
Y-tube choice-tests evaluating the olfactory preferences of *Tribolium confusum* females for rice kernels (5 g) with different infestation status. Thirty responsive beetles were tested for every comparison. For each comparison, χ^2^ and *P* value are provided when a significant difference (P < 0.05) in the number of choosing beetle is recorded. Subscripts reported for χ^2^ represent the degrees of freedom. ns = no significant difference; SZ = *Sitophilus zeamais*, TC = *Tribolium confusum*, CF = *Cryptolestes ferrugineus*; NC = Number of beetle females who performed no choice between the two given cues.

Results highlighted that all the tested olfactory sources were innately appealing for *T. confusum* females (Fig. 1). Indeed, females positively oriented toward both intact and *S. zeamais-*infested rice. Furthermore, the presence of *S. zeamais* adults did not alter *T. confusum* orientation. However, when compared in two-choice Y-tube assays, *S. zeamais-*infested rice was preferred over intact rice alone, as well as over intact rice infested just with *S. zeamais* adults (Fig. 2). The presence of adults of *S. zeamais* in the odorous sources did not affected behavioural responses of *T. confusum* females, since tested specimens did not even discriminate between intact rice and rice with adult maize weevils (Fig. 2). No significant differences in latent periods were recorded (Suplementary Table S1).

*Tribolium confusum* females displayed different responses toward double-infested rice, although both were innately attractive (Fig. 1) and were preferred to intact rice (Fig. 2**)**. Furthermore, the sole presence of either *T. confusum* or *C. ferrugineus* adults in *S. zeamais-*infested rice did not alter the chemotaxis of tested specimens, which did not discriminate the olfactory stimuli of these sources from those of *S. zeamais*-infested rice. Nevertheless, when each double-infested rice (containing larvae of both primary and secondary pests) was evaluated against rice infested by *S. zeamais* larvae alone in two-choice bioassays, *T. confusum* females’ responses were dissimilar. *Tribolium confusum* females showed to recognize self-infestation by choosing rice infested by larvae of *T. confusum* and *S. zeamais* over the single *S. zeamais*-infested rice (Fig. 2). In contrast, in presence of a hetero-specific double infestation, *T. confusum* females preferred *S. zeamais*-infested rice over rice infested by *C. ferrugineus* and *S. zeamais* larvae (Fig. 2). No significant differences in latent periods were recorded (Suplementary Table S1).

### Identification of VOCs from rice-sources

**Figure 3:**
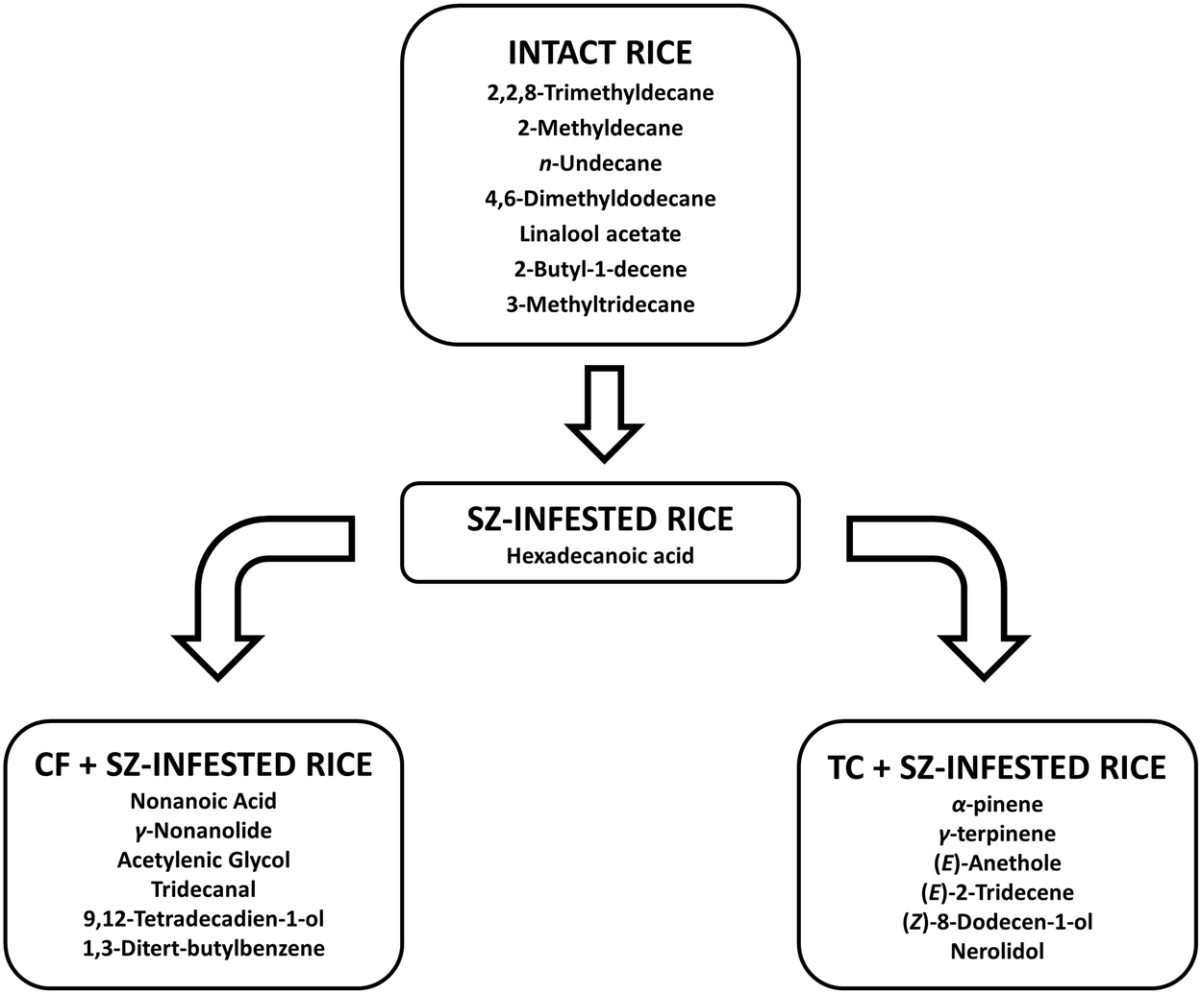
VOCs exclusively produced by tested rice sources according to different infestation status. SZ = *Sitophilus zeamais*, TC = *Tribolium confusum*, CF = *Cryptolestes ferrugineus*.

GC/MS analyses allowed to identify 70 different molecules (Supplementary Table S2). VOC emissions qualitatively varied according to infestation status of rice, highlighting the presence of only 21 compounds commonly produced by all the tested sources. Pest feeding-activity affected the complexity of VOC emission either by directly producing the volatiles (e.g. faeces and pheromones) or by inducing their ex-novo production from plant material. Intact rice kernels emitted a lower number of volatiles (28 VOCs) compared to *S. zeamais*-infested rice (45 VOCs). Furthermore, also the presence of a secondary feeder enriched the volatile bouquet of rice kernels, highlighting 54 and 56 VOCs emitted by *T. confusum* + *S. zeamais*-infested and *C. ferrugines* + *S. zeamais*-infested rice respectively. Overall, the tested rice sources showed characteristic volatile profiles and several VOCs were exclusive of a given infestation status (Fig. 3). Intact rice emitted 7 volatiles which disappeared following pest infestation, while in *S. zeamais*-infested rice were recorded 21 VOCs associated to *S. zeamais* infestation and a single VOC (i.e. hexadecanoic acid) exclusively emitted by this olfactory source. When a double infestation occurred, the production of 6 novel molecules, which can be related to the presence of whatever external feeder, were noted (Supplementary Table S2). However, the feeding activity of each secondary pest could be recognized according to the different volatile emissions. Rice bulks infested simultaneously by *S. zeamais* and *C. ferrugineus* emitted 56 identified VOCs, 6 of which were exclusive of this olfactory source. Similarly, rice double infested by *S. zeamais* and *T. confusum* emitted 6 peculiar VOCs out of a total of 54 volatiles, while 2 molecules were negatively correlated to *T. confusum* feeding-activity (Supplementary Table S2).

**Figure 4:**
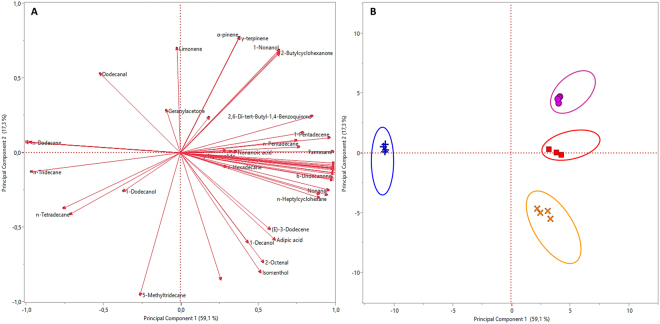
Principal Component Analysis (PCA) of volatile profiles from differentially infested rice. (**A**) PCA loading plot, showing volatile correlations with the first and second principal component; (**B**) PCA score plot, highlighting cluster of volatiles attributable to different infestation status (ellipses = 95% of confidence).  Intact Rice;  *Sitophilus zeamais*-infested rice;  *Tribolium confusum* + *Sitophilus zeamais*-infested rice;  *Cryptolestes ferrugineus* + *Sitophilus zeamais*-infested rice.

PCA followed by multi-factorial analysis (MFA) highlighted differences among volatile emissions from different-infested rice. The Kaiser coefficient was around 1.00, since no correlations among the majorities of the compounds occurred and the variables could be considered independent. Three principal components, explaining 91.70% of variance, were selected (i.e. corresponding to the flex point of the scree plot obtained by PCA) and analysed (Supplementary Table S3). Results from PCA are presented in Fig. 4A and a score plot shows the main sources of variation considering the selected principal components (Fig. 4B). The eigenvectors of principal components (Supplementary Table S4) and of rotated factors (Supplementary Table S5) were calculated for any single identified VOCs. The rotated factors with an eigenvector of at least ± 0.5 were considered for the following analyses, to label each factor according with the involved source of variability (Supplementary Table S5). Based on the results of MFA, the 3 evaluated factors were labelled as: Factor1 “*Sitophylus zeamais*” (explained variance = 54.25%), Factor2 “*Cryptolestes ferrugineus*” (explained variance = 19.66%) and Factor3 “*Tribolium confusum*” (explained variance = 17.88%). Indeed, for every selected factor, pest presence was associated to peculiar VOCs (i.e. those with positive eigenvectors), whereas volatiles showing negative eigenvectors were negatively correlated to infestation. In detail, Factor 1 showed strong correlation with 45 VOCs, while Factor 2 and Factor 3 with 16 VOCs each (Supplementary Table S5).

## Discussion

Pest infestation strongly modifies volatile profiles emitted by rice kernels and alters behavioural responses of *T. confusum* females. According to Ehrlich and Raven^33^, secondary plant substances play a key role in the evolutionary interactions between insects and plants, since their main objective is to attract or repel insects. Our results empathize that the feeding activity of tested species affects the whole volatile profiles of rice grains, promoting or reducing the emission of peculiar VOCs which can be strongly correlated to every pest species. This kind of VOCs have been already acknowledged to act as kairomones toward natural enemies of stored product pests, including several parasitoid species^21,34–36^. For instance, differences among volatile profiles attributable to diverse ongoing infestations may be exploited by either generalist and specialist parassitoids to discriminate and locate their hosts. Here, we identified 1-pentadecene, a component of *T. confusum* larval faeces, which serve as attractant for the parasitoid *Holepyris sylvanidis* (Brèthes) (Hymenoptera: Bethylidae)^37^. Although this molecule is recognised to be directly produced by *T. confusum* larvae, here we highlighted that it is a common VOC recurrent in *S. zeamais-*infested sources. However, emission of 1-pentadecene reached the highest level in rice co-infested by *T. confusum* and *S. zeamais* larvae. Since *S. zeamais* performances may be impaired by hetero-specific larvae feeding on grain external surface, its fitness may profit from the recruitment of *T. confusum* natural enemies, which can benefit from an early host-habitat location.

To date, the effect of distinctive herbivore-induced grain volatiles (HIGVs) on other community members belonging to the same trophic level is still uncertain and poorly investigated. Our results demonstrate that olfactory cues route searching behaviour of *T. confusum* females, allowing them to distinguish host grains with different suitability values by detecting peculiar HIGVs. However, all the tested host-food sources are innately attractive for *T. confusum* and confused beetle females revealed naïve attraction also for intact rice kernels, which may be considered a less suitable resource. The presence of a primary pest has been already proved to increase positive orientation and fitness of various secondary feeders^38^. According to previous investigation on a close related species^39^, here *T. confusum* females prefer *S. zeamais-*infested rice over intact rice, irrespectively to the presence of adult maize weevils. Early infestation (i.e. the presence of adult *S. zeamais* alone) does not affect behavioural responses of *T. confusum*, which is incapable to detect precocious infestations and is unaffected by olfactory cues directly correlated to adult feeding activity. In contrast, the feeding activity of *S. zeamais* larvae induces the emission of 22 VOCs, which can either ex-novo produced by the grain, as indirect defence, as well as directly excreted by the larvae inside the kernels. Among the identified VOCs, only hexadecanoic acid is exclusively correlated to *S. zeamais* infestation, since this molecule disappears when a double infestation occurs. Moreover, hexadecanoic acid has been already recorded as aggregation cue for the females of khapra beetle, *Trogoderma granarium* Everts (Coleottera: Dermestidae), one of the most destructive pests of stored grains and seeds^40^.

Furthermore, the incidence of primary infestation on foodstuff usually promotes the outbreak of fungal infections, which can either change the environmental conditions as well as affect the volatile emissions^41^. Indeed, fungi are able to release a number of VOCs including aliphatic eight-carbon compounds derived from the degradation of unsaturated fatty acids^42^. Several fungi-related VOCs are emitted by *S. zeamais-*infested rice, highlighting that the insurgence of mold infection is favoured when kernels are damaged by an internal feeder. The presence of an external feeder generally reduces the emission of fungal volatiles, since secondary pests consume also fungal mycelia when feeding on kernels^43^. Indeed, as a primary pest of stored grain, *S. zeamais* can contribute to the dispersal of fungal spores and provide through feeding damages entry points for fungal infections^44^. Furthermore, those fungal-related molecules may also impact the other components of trophic contexts, as natural enemies. For instance, the parasitoid *Lariophagus distinguendus* Förster (Hymenoptera: Pteromalidae) use fungi-related volatiles for host-habitat assessment to avoid moldy grains which can be less profitable for the survival of the parasitized larvae^45^.

Population-based competition implies the concept of resource-limitation, which can even occur among conspecifics as scramble (i.e. without behavioural interferences) and contest (i.e. *via* aggressive behaviours) competition. Indeed, individuals of the same species have the same resource requirements, while different species could just present some resource overlaps, thus generally classifying the interspecific competition as less effective than the intraspecific one. Nevertheless, *T. confusum* females show to be innately attractive by rice co-infested by *T. confusum* and *S. zeamais* and even to prefer rice infested by conspecific over singly *S. zeamais-*infested kernels. This behavioural response cannot be attribute to pheromone release by other *T. confusum* adults, since tested females are not able to discern and identify the presence of adult conspecifics within the kernels in choice bioassays. However, *T. confusum* larval infestation cause the emission of 6 characteristic HIGVs, which can act as attractant for conspecific adults. Among these exclusive volatiles, three terpenes have been identified: two monoterpenes, *α-*pinene and *γ*-terpinene, and a sesquiterpene oxygenated, nerolidol, which have been already recorded as attractant for a number of Coleopteran species^46^. Definitely, terpenes are the largest class of natural molecules and are generally devoted to mediate the interactions between organisms, whether toward conspecifics and beneficial species, or as defence response^47^. Remarkably, (*E*)-anethole, a recurrent component of essential oils, has been identified in *T. confusum-*infested rice, although its insecticidal activity has been already investigated against a number of stored product pests^8^. The responses recorded for *T. confusum* in behavioural assays could seem in contrast with the fundamental concept of competition and a kind of maladaptive behaviour. However, as already demonstrated for other stored-product pests^48,49^, these results can highlight the occurrence of an adaptive behaviour that may help *T. confusum* females to readily detect a host-grain source previously colonized by conspecifics and thus certainly suitable for oviposition and larval development. Indeed, the ecological consequence of intraspecific interactions need to be carefully evaluated by each individual^50^, since competitive outcomes depend on the availability of resources, population density, developmental requirements and life-history traits of every species^51^. Thus, when competing for local resources, individuals of the same species develop adaptive responses just to overcome extremely harmful situations, which can lead to serious negative fitness consequences^52,53^.

According to niche theory, interspecific competition is mainly attributable to the overlap of the resources exploited by the involved species^54^. However, the coexistence of two species sharing the same trophic niche can take place and hetero-specific interactions can lead to different competitive results, according to population density and colonization order^55^. Furthermore, to avoid interference with aggressive species, many insects evolved conflict-avoidance behaviours to recognize chemically marked resources, which have been already exploited and colonized^56^. Avoidance of direct conflict is favourable for both dominant and minor species, since interference competition involving aggressive behaviours is energetically expensive and can cause severe mortality also for the predominant species^14,25,57^. Plant-mediated interactions are frequent among contending species, and can involve the induction of chemical responses in the host-plant or grain to deter competitor feeding activity^58,59^. Indirect plant-mediated competition is consistent with the behavioural responses of *T. confusum* females toward rice co-infested by *S. zeamais* and *C. ferrugineus* larvae. It has been already investigated the negative impact of *C. ferrugineus* on the development and survival of other stored-product Coleoptera, including *Tribolium castaneum* Herbst (Coleoptera: Tenebrionidae), a close related species of *T. confusum*^13,15^. Consistently with previously described results, confused beetle females are not able to detect *C. ferrugineus* early infestation, whereas the presence of larval stages of rusty grain beetles evokes negative chemotaxis. Feeding activity of hetero-specific larvae induce the emission of 6 peculiar HIGVs, which may affect the orientation of *T. confusum* females and serve as repellents. In detail, nonanoic acid and γ-nonanolide are recognized allelochemicals among Coleoptera, while 9,12-Tetradecadien-1-ol is a pheromonal component of many pyralid moths, including the foodstuff pests *Ephestia kuehniella* Zeller^60^ and *Plodia interpunctella* Hübner (Lepidoptera: Pyralidae)^61,62^.

Our results clearly reveal that olfactory stimuli can route interactions between members of the same guild and thus play a role on the coexistence and the competition establishing among stored-product pests. Furthermore, the feeding activity of tested species impacts the whole volatile profiles of rice grains, promoting or reducing the emission of specific VOCs which can be strongly correlated to every pest species infestation. To the best of our knowledge, this is the first time that the occurrence of indirect plant-mediated competition has been demonstrated for granivorous insects. Overall, deep knowledge of the mechanisms underlying interspecific interactions and associations among stored-product pests is pivotal to develop and design new and effective pest-control strategies. Therefore, the identified putative attractants and repellents specific for *T. confusum* could represent an innovative tool to enhance Integrated Pest Management (IPM) against stored-product pests.

## Materials and Methods

### Grain and insect materials

Paddy rice kernels (var. Ribe, supplied by a local producer and organic certified) were used as rearing medium for all the insects involved in the experimental set up. Prior to use rice grain as rearing medium, the presence and the insurgence of previous infestations was checked for 30 days, to avoid possible kernel contaminations from other insect pests. Insect species were individually reared on rice medium for at least three generations. *Sithopilus zeamais* was reared using intact rice kernels, while *T. confusum* and *C. ferrugineus* were fed with mechanically crushed grains. Insect rearing were placed in a climatic chamber and maintained under controlled conditions [25 ± 1 °C, 50 ± 5% RU and 12:12 (light:dark) photoperiod]. Adult insects (*S. zeamais*, *T. confusum* and *C. ferrugineus*) from these rearing media were used to infest the rice employed for the bioassays or the volatile collection.

Four different rice-based sources were then set up: (*i*) intact rice, (*ii*) *S. zeamais-*infested rice (SZ-infested rice), (*iii*) *T. confusum* and *S. zeamais* double-infested rice (TC + SZ-infested rice), (*iv*) *C. ferruginues* and *S. zeamais* and double-infested rice (CF + SZ infested rice). To obtain *S. zeamais-*infested rice, 50 unsexed adult maize weevils (15–30 days old) were placed in a glass vessel (1 L volume) containing 500 g of intact rice to allow feeding and oviposition for 5 days. Then, *S. zeamais* adults were removed and infested rice was stored at controlled conditions for 23–25 days, to allow larval growth inside the kernels, before been tested. Double-infested rice was similarly obtained inserting 50 adults *S. zeamais* (15–30 days old) in an analogous glass vessel with 500 g of intact rice. After 5 days *S. zeamais* adults were removed and the infested rice was kept for 7 days without adult insects. Then, 50 unsexed *T. confusum* or *C. ferrugineus* (15–30 days old) were introduced in the medium and allowed to infest the grains for 5 days, before being removed. Lastly, the obtained double-infested rice-media were stored for 12–14 days at controlled conditions, prior to be used in the experimental set up. Intact rice (500 g) was also placed in a 1L-glass vial and maintained at the same conditions of infested rice-bulks for 25–30 days Before been employed for the behavioural assays or the volatile collection, all the tested rice-based sources were maintained at the above mentioned controlled conditions for the entire duration of trials.

To avoid previous experience, and thus conditioning to volatiles emitted by rice kernels, *T. confusum* females, which were tested in the bioassays, fed on a different rearing medium (i.e. wheat flour) for at least three generations. Insect rearing were placed in a climatic chamber and maintained under controlled conditions during the entire experimental duration [25 ± 1 °C, 50 ± 5% RU and 12:12 (light:dark) photoperiod].

### Olfactometry

All experiments were conducted at 25 ± 1 °C (45–55% R.H.); to avoid visual cues, all the trials were carried out in a dark plastic box (1.5 × 1 × 0.7 m) illuminated with red fluorescent tubes (20 W). The bioassays were conducted in a Y-tube olfactometer connected with an air delivery system which blown the air at 0.5 L min^−1^ constant flow. The inflow was purified by passing through an activated charcoal filter. The Y-tube system was a horizontal glass unit consisting of a central tube (100 × 30 mm diam) and two lateral arms (90 × 30 mm diam) ending with a spherical chamber (50 mm diam). Two Drechsel bottles (250 mL), containing odorous sources, were connected to the lateral arms with Teflon connections. Equally, previously humidified and purified air was blown into the Drechsel bottles through Teflon connections.

*Tribolium confusum* females were tested at 14–21 days of age and were used only once. To exclude the presence of visual cues affecting insect orientation, preliminary trials were performed using no odorant sources (blank vs blank), showing no positional effect. However, replicates of every treatment were conducted over several days (to allow for a daily variability in responsiveness) and the olfactometer arms were reversed after every test to control for any other positional effect. The olfactometer was washed with warm water (35–40 °C) and mild soap every 5 replicates, and then rinsed with hot water, followed by distilled water. A total of 30 responsive *T. confusum* females were tested for each comparison, with every specimen gently introduced into the central arm of the Y-tube from a glass vial and observed for 6 min. Individuals that remained unresponsive for 5 min were discarded and labelled as no-choice. For insects that started searching within 5 min, a choice was recorded when the female remained in one of the Y-tube arms for at least 20 seconds. For each replicate, we recorded the latent period (i.e. time spent inside the choice arena before entering an arm) and the choice (i.e. the odor source selected).

### Behavioural responses of Tribolium confusum females toward different rice-sources

The following bioassays were set up to investigate: (*i*) the attractiveness toward *T. confusum* females of intact rice and rice infested by primary and/or secondary pests, (*ii*) the naïve preferences for intact and *S. zeamais-*infested grain and (*iii*) the alteration of those preferences attributable to the occurrence of a secondary infestation (*T. confusum* or *C. ferrugineus*).

(i) To assess the naïve attractiveness of grain with different infestation status, the following treatments were tested against blank in Y-tube bioassays: a) intact rice; b) intact rice plus 10 adult *S. zeamais*; c) SZ*-*infested rice; d) SZ*-*infested rice plus 10 adult *S. zeamais;* e) TC + SZ-infested rice; f) CF + SZ-infested rice. Treatments b) and d) were obtained placing 10 adult maize weevils in 5 g of intact and SZ*-*infested rice respectively 24 h before being used for olfactometer trials. This incubation time (24 h) was selected to allow insect feeding activity, odour contamination (also from faeces) and possibly oviposition activity, while preventing the presence of newly-born larvae, which could alter the emission by feeding on the kernels. Five grams of every odour source were tested for each comparison.
(ii) To assess the impact of a primary infestation on *T. confusum* preferences, two-choice Y-tube bioassays were performed by pair-comparing the following treatment: a) intact rice; b) intact rice plus 10 adult *S. zeamais*; c) SZ*-*infested rice; d) SZ*-*infested rice plus 10 adult *S. zeamais*. All possible binary combinations were carried out. The experiment aimed to explore the effect of S*. zeamais* presence (both as adult and/or larvae) on the appeal of food-sources. Five grams of every odour source were tested for each comparison.
(iii) The effect of larval infestation by a secondary pest was evaluated in two choice bioassays against intact and SZ*-*infested rice. To evaluate the effect of an early infestation of either secondary pests (*T. confusum* or *C. ferrugineus*), the chemotaxis of confused beetle females toward SZ*-*infested rice plus secondary pest adults (i.e. 10 adult *T. confusum* or *C. ferrugineus* added in 5 g SZ*-*infested rice 24 h prior to test) was evaluated against SZ-infested rice and double-infested rice in two-choice bioassays. This incubation time (24 h) was selected to allow insect feeding activity, odour contamination (also from faeces) and possibly oviposition activity, while preventing the presence of newly-born larvae, which can alter the emission by feeding on the kernels.

For every described comparison, binary choice data were analyzed by χ^2^ likelihood test with Yates’ correction (α = 0.05); latent periods were analyzed in JMP 11^®^ using a generalized linear model with ‘treatment’ as a fixed factor, after verifying normality distribution and variance homoscedasticity by Shapiro–Wilk and Levene’s test respectively.

### VOC collection and identification

Head-space samplings of the paddy rice were performed using a Supelco^®^ (Bellefonte, PA, USA) SPME device coated with polydimethylsiloxane (PDMS, 100 μm), which can selectively extract the majority of secondary metabolites, acting as infochemicals for insects. Equilibration and SPME sampling was achieved using the same new fiber, preconditioned according to the manufacturer instructions. For all treatments, 5 g of intact or infested rice were inserted into a 20-mL hermetic glass vial, and allowed to equilibrate for 30 min. After the equilibration time, the fiber was inserted in the vial and exposed to the headspace for 30 min. Samplings were accomplished in a thermostatic chamber (25 ± 1 °C) to guarantee the stable temperature. Once samplings were completed, the fiber was withdrawn into the needle and transferred to the injection port of the GC/MS system. According to the results of behavioural assays, the following treatment were evaluated: a) intact rice; b) SZ-infested rice; c) TC + SZ-infested rice and d) CF + SZ-infested rice. Four replicates (i.e. each containing 5 g of rice) for every treatment were performed. All the SPME sampling and desorption conditions were identical for all the samples and replicates. Blanks were performed before first SPME extraction and repeated randomly during each series. Relative-quantitative comparisons of peak areas were provided for the same identified chemical in different samples, after verifying that the intensity of the chromatograms was equivalent.

GC/MS analyses were performed with a Thermo Fisher TRACE 1300 gas chromatograph equipped with a MEGA-5 capillary column (30 m x 0.25 mm; coating thickness = 0.25 μm) and a Thermo Fisher ISQ LT ion trap mass detector (emission current: 10 microamps; count threshold: 1 count; multiplier offset: 0 volts; scan time: 1.00 second; prescan ionization time: 100 microseconds; scan mass range: 30–300 m/z; ionization mode: EI). The following analytical conditions were employed: injector and transfer line temperature at 250 and 240 °C, respectively; oven temperature programmed from 60 to 240 °C at 3 °C min^−1^; carrier gas, helium at 1 mL min^−1^; splitless injection. Identification of chemicals was based on the comparison of retention times (RT) and their linear retention indices (LRI), relative to the series of n-hydrocarbons, with those of pure chemicals, and on computer matching against the commercial libraries (NIST 98 and ADAMS) and compared to a homemade library built from pure substances, components and the MS literature data^63–65^. LRI was calculated by comparing the retention times of the compounds to those of a standard mixture of alkanes (C7-C30 saturated alkanes standard mixture, Supelco^®^, Bellefonte, PA, USA), which were sampled with the same fiber and the same procedure described for rice, as well as was analysed by GC/MS set at the identical conditions of biological samples.

To compare relative volatile emissions among different-infested rice, the variance between peak areas was analysed using JMP 11^®^ for each compound emitted by two or more tested treatments. Before statistical analysis, the area integration report of every identified compound was transformed into Log values. Normality distribution and variance homoscedasticity were verified by the Shapiro–Wilk and Levene’s test respectively. Differences in volatile emissions among different treatments were analysed using a general linear model with a normal error structure and one fixed factor: y = Xß + ε where y is the vector of the observations, X is the incidence matrix, ß is the vector of fixed effect (i.e. treatment), and ε is the vector of the random residual effects. We used the False Discovery Rate^66^ to control for the experiment-wide error rate inherent in large numbers of comparisons. Principal Component Analysis (PCA), followed by Multi-Factorial Analysis (MFA), was performed on transformed values of each VOCs using JMP 11^®^ software. MFA was achieved using a principal component procedure and a VARIMAX orthogonal rotation technique, and the scores of common factors were calculated^67^.

### Data availability

The datasets generated and/or analysed during the current study are available from the corresponding author on reasonable request.

## Electronic supplementary material

Supplementary Files
