# Supplementary material for: VOC emissions influence intra- and interspecific interactions among stored-product Coleoptera in paddy rice
Source: Sci Rep. 2018 Feb 1;8:2052. doi: 10.1038/s41598-018-20420-2 (PMC5794852; doi:10.1038/s41598-018-20420-2)
Supplement: Supplementary file 1 — Supplementary Files [file 41598_2018_20420_MOESM1_ESM.pdf]

VOC emissions influence intra- and interspecific interactions among stored-product Coleoptera in paddy rice

Giulia Giunti[@], Vincenzo Palmeri, Giuseppe Massimo Algeri, Orlando Campolo

Department of Agriculture, University "Mediterranea" of Reggio Calabria, Loc. Feo di Vito, 89122 Reggio Calabria, Italy

[@] Corresponding author.

Address: Loc. Feo di Vito, 89122 Reggio Calabria, Italy

tel: +39 0965 1694266; e-mail address: giulia.giunti@unirc.it; giunti.giulia@gmail.com

Supplementary Table S1 Latency time displayed before choice each given odour source (i.e. Treatments A and B) by *Tribolium confusum* females during bioassays in Y-tube olfactometer. Thirty responsive beetles were tested for each comparison. A generalized linear model was employed to highlight significant differences between the treatments of every tested comparison.

ns = no significant difference

**Treatment A**			**Treatment B**					
**Olfactory Cue**	**N**	**Latent Period ± SE (s)**	**Olfactory Cue**	**N**	**Latent Period ± SE (s)**	$\chi^2_{1,28}$	***P value***	
Intact Rice	25	110.56 ± 17.71	Blank	5	140.80 ± 53.92	0.4603	0.4975	ns
SZ-infested Rice	23	101.04 ± 17.02	Blank	7	54.86 ± 20.15	2.0281	0.1544	ns
Intact Rice + SZ adults	22	114.05 ± 18.31	Blank	8	107.00 ± 28.12	0.0438	0.8342	ns
SZ-infested Rice + SZ adults	22	106.14 ± 14.13	Blank	8	141.50 ± 34.09	1.3672	0.2423	ns
TC&SZ-infested Rice	23	149.70 ± 19.93	Blank	7	106.00 ± 20.92	1.3691	0.2420	ns
CF&SZ-infested Rice	21	117.57 ± 16.30	Blank	9	97.44 ± 25.33	0.4263	0.5138	ns
Intact Rice	6	167.67 ± 38.66	SZ-infested Rice	24	158.67 ± 19.58	0.0454	0.8312	ns
Intact Rice	13	131.15 ± 22.11	Intact Rice + SZ adults	17	146.12 ± 21.78	0.2401	0.6241	ns
Intact Rice	8	126.25 ± 33.09	SZ-infested Rice + SZ adults	22	140.45 ± 22.22	0.1224	0.7264	ns
Intact Rice + SZ adults	8	109.38 ± 34.02	SZ-infested Rice	22	70.95 ± 12.02	0.0161	0.8991	ns
Intact Rice + SZ adults	7	112.71 ± 30.06	SZ-infested Rice + SZ adults	23	68.65 ± 12.24	2.6302	0.1048	ns
SZ-infested Rice	15	129.00 ± 22.56	SZ-infested Rice + SZ adults	15	125.13 ± 22.08	0.0161	0.8991	ns
TC&SZ-infested Rice	23	103.09 ± 17.01	Intact Rice	7	82.86 ± 22.18	0.3917	0.5314	ns
TC&SZ-infested Rice	22	104.82 ± 18.38	SZ-infested Rice	8	88.38 ± 24.07	0.2514	0.6161	ns
TC&SZ-infested Rice	22	117.45 ± 20.69	SZ-infested Rice + TC adults	8	159.75 ± 27.55	1.2830	0.2573	ns
SZ-infested Rice	14	61.14 ± 7.56	SZ-infested Rice + TC adults	16	89.25 ± 23.36	1.2264	0.2681	ns
CF&SZ-infested Rice	23	132.52 ± 16.53	Intact Rice	7	84.86 ± 16.49	2.3501	0.1253	ns
CF&SZ-infested Rice	9	77.89 ± 17.95	SZ-infested Rice	21	115.33 ± 18.48	2.8229	0.0806	ns
CF&SZ-infested Rice	9	140.22 ± 26.80	SZ-infested Rice + CF adults	21	141.00 ± 19.33	0.0005	0.9813	ns
SZ-infested Rice	15	89.20 ± 15.30	SZ-infested Rice + CF adults	15	129.40 ± 19.99	2.3710	0.1231	ns

Supplementary Table S2 Volatiles emitted by rice sources with different infestation status. Peak area values are provided as mean ± standard error of four replicates. Statistical values (F ratio and *P* value) are specified for each single chemical. Different letters represent significant differences between the values of the same row (Tukey's HSD). LRI was calculated according to retention times of sutured alkanes (C7-C30).

SZ= *Sitophilus zeamais*; TC= *Tribolium confusum*; CF= *Cryptolestes ferrugineus*.

LRI= Linear Retention Index

VOC emissions	LRI	Chemical	Intact Rice	SZ-infested Rice	TC+SZ-infested Rice	CF+SZ-infested Rice	$F_{3,12}$	*P*
Exclusive Intact Rice	1024	2,2,8-Trimethyldecane	27323.51 ± 4786.71	0.00 ± 0.00	0.00 ± 0.00	0.00 ± 0.00		
	1054	2-Methyldecane	34266.07 ± 5931.32	0.00 ± 0.00	0.00 ± 0.00	0.00 ± 0.00		
	1101	Undecane	40039.39 ± 4756.61	0.00 ± 0.00	0.00 ± 0.00	0.00 ± 0.00		
	1212	4,6-Dimethyldodecane	26816.24 ± 2518.67	0.00 ± 0.00	0.00 ± 0.00	0.00 ± 0.00		
	1258	Linalool acetate	19422.06 ± 4129.30	0.00 ± 0.00	0.00 ± 0.00	0.00 ± 0.00		
	1367	2-Butyl-1-decene	12626.96 ± 1155.47	0.00 ± 0.00	0.00 ± 0.00	0.00 ± 0.00		
	1371	3-Methyltridecane	14731.78 ± 826.66	0.00 ± 0.00	0.00 ± 0.00	0.00 ± 0.00		
SZ-infested Rice	907	Heptanal	0.00 ± 0.00 b	49266.61 ± 21225.72 a	23995.55 ± 5271.78 a	20759.56 ± 2459.42 a	383.05	<.0001*
	956	(E)-2-Heptanal	0.00 ± 0.00 c	75227.98 ± 12679.76 a	37017.90 ± 11647.89 b	35540.61 ± 1510.48 b	2303.21	<.0001*
	985	Hexanoic acid	0.00 ± 0.00 c	548206.73 ± 144455.59 a	286181.53 ± 29535.46 ab	185999.32 ± 30437.37 b	3026.88	<.0001*
	1003	Octanal	0.00 ± 0.00 c	653678.05 ± 88436.62 a	340283.52 ± 28193.39 b	331020.65 ± 25298.62 b	10213.97	<.0001*
	1038	3-Octen-2-one	0.00 ± 0.00 d	1364691.66 ± 89212.01 a	625139.50 ± 12467.77 b	516036.12 ± 35148.95 c	37391.84	<.0001*
	1074	1-Octanol	0.00 ± 0.00 c	284779.49 ± 30773.99 a	88968.46 ± 10439.41 b	74705.20 ± 8790.27 b	6561.61	<.0001*
	1098	2,5-Dimethylcyclohexanol	0.00 ± 0.00 c	223926.92 ± 22763.56 a	122667.04 ± 8267.54 b	116424.12 ± 4350.42 b	15336.13	<.0001*
	1179	Octanoic acid	0.00 ± 0.00 b	106820.09 ± 63914.20 ab	632.13 ± 102.38 ab	9252.11 ± 4668.21 a	5.09	0.019*
	1214	(2E,4E)-2,4-Nonadienal	0.00 ± 0.00 c	158100.15 ± 13827.80 a	90706.54 ± 3657.82 b	94154.51 ± 11351.16 b	12227.68	<.0001*
	1262	(E)-2-Decenal	0.00 ± 0.00 b	137684.11 ± 91609.88 a	41249.74 ± 2563.87 a	46591.95 ± 3828.25 a	723.4	<.0001*
	1274	6-Undecanone	0.00 ± 0.00 c	72533.05 ± 21192.52 a	10413.79 ± 1098.93 b	10176.18 ± 1419.19 b	1582.06	<.0001*
	1280	2,6,11-Trimethyldodecane	0.00 ± 0.00 b	50039.83 ± 2915.05 a	10275.82 ± 2315.24 a	14291.57 ± 1693.54 a	2249.15	<.0001*
	1290	Pentyl hexanoate	0.00 ± 0.00 c	42079.54 ± 8478.91 a	13700.97 ± 640.77 b	9757.82 ± 1100.15 b	3419.97	<.0001*
	1307	Undecanal	0.00 ± 0.00 c	52686.95 ± 6540.33 a	14768.75 ± 845.75 b	14266.57 ± 1694.09 b	6205.13	<.0001*
	1320	(Z)-2-Tridecene	0.00 ± 0.00 b	74700.99 ± 8571.68 a	62350.80 ± 6256.79 a	47631.61 ± 9636.69 a	3434.83	<.0001*
	1326	Methyl decanoate	0.00 ± 0.00 c	95856.82 ± 4222.35 a	13547.87 ± 1259.44 b	18395.71 ± 4617.07 b	3158.63	<.0001*
	1336	Farnesane	0.00 ± 0.00 d	62473.53 ± 5439.93 a	341358.77 ± 23385.10 b	13948.15 ± 1424.22 c	10689.54	<.0001*

VOC emissions	LRI	Chemical	Intact Rice	SZ-infested Rice	TC+SZ-infested Rice	CF+SZ-infested Rice	$F_{3,12}$	*P*
	1362	1-Undecanol	0.00 ± 0.00 b	84504.74 ± 38169.76 a	59507.31 ± 3906.38 a	27249.03 ± 8061.20 a	436.5	<.0001*
	1375	2-Butyl-2-octenal	0.00 ± 0.00 b	45032.65 ± 6345.80 a	31685.76 ± 3675.57 a	28205.53 ± 10277.53 a	1561.62	<.0001*
	1480	(E)-2-Dodecen-1-ol	0.00 ± 0.00 c	38778.05 ± 3715.11 a	23329.28 ± 3659.87 a	5416.06 ± 1384.01 b	2031.13	<.0001*
	1492	1-Pentadecene	0.00 ± 0.00 d	47151.22 ± 7245.11 b	1753070.10 ± 167811.00 a	6245.85 ± 1762.96 c	1934.4	<.0001*
Exclusive SZ-infested Rice	1969	Hexadecanoic acid	0.00 ± 0.00	353284.48 ± 345628.00	0.00 ± 0.00	0.00 ± 0.00		
Double-infested Rice	1090	1-Undecene	0.00 ± 0.00 c	0.00 ± 0.00 c	48383.52 ± 7077.97 a	23816.24 ± 2269.15 b	8278.76	<.0001*
	1124	2-Ethylhexanoic acid	0.00 ± 0.00 b	0.00 ± 0.00 b	24343.01 ± 4405.83 a	28808.36 ± 8321.10 a	2045.47	<.0001*
	1166	1-Nonanol	0.00 ± 0.00 a	0.00 ± 0.00 a	21407.39 ± 4559.71 b	12755.59 ± 1437.44 b	3348.94	<.0001*
	1259	γ-Octanolactone	0.00 ± 0.00 b	0.00 ± 0.00 b	13704.54 ± 1458.70 a	14717.54 ± 1452.30 a	13651.74	<.0001*
	1268	2-Butylcyclohexanone	0.00 ± 0.00 b	0.00 ± 0.00 b	33365.71 ± 4684.80 a	35538.38 ± 4812.94 a	6718.12	<.0001*
	1348	10-Methyl-trans-2-decalone	0.00 ± 0.00 b	0.00 ± 0.00 b	28057.75 ± 3929.61 a	43011.36 ± 5837.28 a	7409.83	<.0001*
Exclusive TC&SZ-infested Rice	931	α-pinene	0.00 ± 0.00	0.00 ± 0.00	30234.00 ± 7585.46	0.00 ± 0.00		
	1059	γ-terpinene	0.00 ± 0.00	0.00 ± 0.00	274753.24 ± 51753.96	0.00 ± 0.00		
	1287	(E)-Anethole	0.00 ± 0.00	0.00 ± 0.00	18212.56 ± 1217.25	0.00 ± 0.00		
	1306	(E)-2-Tridecene	0.00 ± 0.00	0.00 ± 0.00	17073.90 ± 1233.10	0.00 ± 0.00		
	1474	(Z)-8-Dodecen-1-ol	0.00 ± 0.00	0.00 ± 0.00	15830.85 ± 2966.08	0.00 ± 0.00		
	1538	Nerolidol	0.00 ± 0.00	0.00 ± 0.00	22040.49 ± 3422.48	0.00 ± 0.00		
Absent TC&SZ-infested Rice	1058	2-Octenal	0.00 ± 0.00 b	88281.33 ± 14733.55 a	0.00 ± 0.00 b	81776.08 ± 5713.35 a	9246.21	<.0001*
	1174	Isomenthol	0.00 ± 0.00 c	164430.81 ± 13596.50 a	0.00 ± 0.00 c	7527.91 ± 1866.65 b	3942.3	<.0001*
Exclusive CF&SZ-infested Rice	1257	1,3-Ditert-butylbenzene	0.00 ± 0.00	0.00 ± 0.00	0.00 ± 0.00	5787.60 ± 328.78		
	1275	Nonanoic Acid	0.00 ± 0.00	0.00 ± 0.00	0.00 ± 0.00	15598.84 ± 3167.90		
	1363	γ-Nonanolide	0.00 ± 0.00	0.00 ± 0.00	0.00 ± 0.00	43445.08 ± 7963.43		
	1419	Acetylenic Glycol	0.00 ± 0.00	0.00 ± 0.00	0.00 ± 0.00	248725.05 ± 228712.93		
	1530	Tridecanal	0.00 ± 0.00	0.00 ± 0.00	0.00 ± 0.00	96882.56 ± 16480.66		
	1642	9,12-Tetradecadien-1-ol	0.00 ± 0.00	0.00 ± 0.00	0.00 ± 0.00	5403.10 ± 843.11		
Common	994	1-Octen-3-ol	34263.29 ± 2890.64 c	303439.80 ± 25579.19 a	191192.45 ± 21676.95 b	132324.18 ± 6636.80 b	121.5	<.0001*
	1029	Limonene	140799.04 ± 13338.85 b	68849.68 ± 19403.34 bc	938263.98 ± 310827.01 a	31235.02 ± 2702.88 c	47.8	<.0001*
	1106	Nonanal	43933.40 ± 7819 c	1250027.42 ± 45340.26 a	647528.77 ± 31569.31 b	725009.71 ± 35371.88 b	291.17	<.0001*
	1193	(E)-3-Dodecene	37393.95 ± 2667.07 b	109692.23 ± 22412.40 a	60280.71 ± 5135.38 ab	52555.33 ± 5405.74 b	10.34	0.001*

VOC emissions	LRI	Chemical	Intact Rice	SZ-infested Rice	TC+SZ-infested Rice	CF+SZ-infested Rice	$F_{3,12}$	*P*
	1200	Dodecane	587393.18 ± 39373.48 a	33581.50 ± 6876.04 b	32502.29 ± 3485.44 b	16480.43 ± 1308.69 c	144.25	<.0001*
	1206	Decanal	25860.53 ± 1481.61 c	102702.13 ± 6407.26 a	73775.57 ± 1976.76 b	60912.62 ± 3812.84 b	125.41	<.0001*
	1242	1-Decanol	14677.15 ± 1194.82 b	31044.26 ± 3107.53 a	18662.56 ± 414.22 b	15550.19 ± 1161.71 b	18.86	<.0001*
	1295	1-Tridecene	17833.29 ± 12933.44 b	38893.12 ± 9061.77 ab	65991.41 ± 4578.12 a	56168.12 ± 6470.63 a	7.23	0.005*
	1300	Tridecane	860372.84 ± 88205.44 a	67211.72 ± 405.99 b	31187.78 ± 2107.98 c	18957.64 ± 1496.39 d	551.07	<.0001*
	1341	n-Heptylcyclohexane	9566.97 ± 703.44 c	577548.30 ± 74986.25 a	230405.13 ± 22687.13 b	242048.32 ± 35156.27 b	221.9	<.0001*
	1354	5-Methyltridecane	13828.07 ± 1085.61 b	72786.90 ± 18553.13 a	605.73 ± 65.51 c	7252.73 ± 2026.73 b	97.04	<.0001*
	1392	1-Tetradecene	41768.62 ± 6114.92 a	25592.57 ± 4558.10 a	10959.68 ± 1046.65 b	7393.74 ± 860.23 b	31.51	<.0001*
	1399	Tetradecane	99655.62 ± 16614.85 a	69987.09 ± 11398.22 a	31506.10 ± 3200.88 b	22292.48 ± 1972.89 b	24.53	<.0001*
	1403	Dodecanal	17645.05 ± 1511.00 a	3087.85 ± 1551.34 b	7612.97 ± 1388.15 ab	9021.69 ± 2183.04 a	7.71	0.004*
	1447	1-Dodecanol	16951.62 ± 3142.68 a	17356.54 ± 1923.61 a	13402.75 ± 3177.54 a	6332.52 ± 646.69 b	8.16	0.003*
	1454	Geranylacetone	15493.74 ± 4536.94	11721.14 ± 3555.01	16939.29 ± 5158.24	11333.09 ± 2234.77	0.5	0.692
	1467	2,6-Di-tert-Butyl-1,4-Benzoquinone	1683.88 ± 323.63 c	5837.12 ± 843.91 ab	10955.85 ± 451.17 a	4989.37 ± 643.89 b	22.79	<.0001*
	1500	Pentadecane	5569.41 ± 1140.09 c	22643.41 ± 2507.93 ab	30269.51 ± 5287.58 a	11887.10 ± 2553.26 bc	16.03	0.0002*
	1541	Hexanedioic acid, di-2-propenyl ester	7633.86 ± 1725.70 b	36222.07 ± 5298.16 a	13077.03 ± 2600.88 b	18644.28 ± 3653.03 ab	8.54	0.003*
	1592	1-Tridecanol	7616.29 ± 1218.70	7698.87 ± 976.69	11782.86 ± 2130.92	9828.17 ± 3447.97	0.58	0.639
	1599	Hexadecane	17207.18 ± 1953.12	24114.20 ± 3145.57	22812.80 ± 2539.10	22695.84 ± 7324.30	0.54	0.666

Supplementary Table S3 Principal components identified after Principal Component Analysis (PCA) of volatile emissions from differentially infested rice.

Number	Eigenvalue	Percentage	Cumulative Percentage
**1**	41.4049	59.150	59.150
**2**	12.0935	17.276	76.426
**3**	10.6917	15.274	91.700
**4**	2.1422	3.060	94.761
**5**	1.1854	1.693	96.454
**6**	0.6504	0.929	97.383
**7**	0.6002	0.857	98.241
**8**	0.3881	0.554	98.795
**9**	0.2798	0.400	99.195
**10**	0.2030	0.290	99.485
**11**	0.1599	0.228	99.713
**12**	0.1334	0.191	99.904
**13**	0.0939	0.134	100.038
**14**	0.0744	0.106	100.144
**15**	0.0321	0.046	100.190
**16**	0.0203	0.029	100.219

Supplementary Table S4 Eigenvectors of every volatile compound emitted by rice sources for 3 selected Principal Components.

PC = Principal Component

**Compound**	**PC1**	**PC2**	**PC3**
Heptanal	0,15426	-0,02602	0,01148
α-pinene	0,05899	0,22207	0,15352
(E)-2-Heptanal	0,15399	-0,03323	0,01127
Hexanoic acid	0,15415	-0,02891	0,0177
1-Octen-3-ol	0,13897	-0,07936	0,09159
Octanal	0,15432	-0,02954	0,01109
2,2,8-Trimethyldecane	-0,15471	0,02066	-0,00484
Limonene	-0,00322	0,20186	0,2041
3-Octen-2-one	0,15406	-0,03097	0,01394
2-Methyldecane	-0,15469	0,02086	-0,0051
2-Octenal	0,08307	-0,21062	-0,12741
γ-terpinene	0,059	0,22207	0,1535
1-Octanol	0,15357	-0,03797	0,01828
1-Undecene	0,09909	0,19828	-0,10147
2,5-Dimethylcyclohexanol	0,1543	-0,02945	0,01107
n-Uncedane	-0,15472	0,02062	-0,00496
Nonanal	0,14885	-0,07154	0,02274
2-Ethylhexanoic acid	0,09904	0,19341	-0,11097
1-Nonanol	0,09909	0,19715	-0,10437
Isomenthol	0,08068	-0,2303	-0,08868
Octanoic acid	0,11654	0,0242	-0,03929
(E)-3-Dodecene	0,08961	-0,14682	0,13041
n-Dodecane	-0,15042	0,02018	0,05589
Decanal	0,13898	-0,08623	0,09354
4,6-Dimethyldodecane	-0,15472	0,02073	-0,00531
(2E,4E)-2,4-Nonadienal	0,15437	-0,02897	0,00975
1-Decanol	0,06769	-0,1724	0,17377
1,3-Ditert-butylbenzene	0,05559	0,00224	-0,28291
Linalool acetate	-0,15468	0,02049	-0,00471
γ-Octanolactone	0,09909	0,19362	-0,11138
(E)-2-Decenal	0,1542	-0,03121	0,0104
2-Butylcyclohexanone	0,09912	0,19362	-0,11054
6-Undecanone	0,15235	-0,05068	0,02391
Nonanoic acid	0,05568	0,00208	-0,28184
2,6,11-Trimethyldodecane	0,15274	-0,04794	0,01353
(E)-Anethole	0,059	0,22196	0,15342
Pentyl hexanoate	0,15331	-0,03851	0,02306
1-Tridecene	0,12288	0,03874	0,00776
n-Tridecane	-0,14957	-0,03654	0,06991
(E)-2-Tridecene	0,059	0,22199	0,15332
Undecanal	0,15333	-0,04114	0,01831
(Z)-2-tridecene	0,15461	-0,02385	0,01391

Methyl decanoate	0,15231	-0,052	0,01912
Farnesane	0,15314	0,00297	0,04863
n-Heptylcyclohexane	0,1471	-0,08078	0,04215
10-Methyl-trans-2-decalone	0,09906	0,19124	-0,11498
5-Methyltridecane	-0,04033	-0,27323	-0,01339
1-Undecanol	0,15415	-0,01987	0,0234
γ-Nonanolide	0,05567	0,00211	-0,28213
2-Butyl-1-decene	-0,15472	0,02062	-0,00502
3-Methyltridecane	-0,15474	0,02078	-0,00519
2-Butyl-2-octenal	0,15442	-0,02671	0,01511
1-Tetradecene	-0,11684	-0,10644	0,14031
n-Tetradecane	-0,11011	-0,11763	0,14577
Dodecanal	-0,08037	0,15258	-0,07834
Acetylenic Glycol	0,05079	0,00261	-0,27496
1-Dodecanol	-0,05689	-0,07378	0,22994
Geranylacetone	-0,01384	0,08186	0,05357
2,6-Di-tert-Butyl-1,4-Benzoquinone	0,13217	0,07066	0,09105
(Z)-8-Dodecen-1-ol	0,05898	0,22178	0,15364
(E)-2-Dodecen-1-ol	0,15272	-0,03031	0,04118
1-Pentadecene	0,14943	0,02887	0,07681
n-Pentadecane	0,11894	0,0113	0,158
Tridecanal	0,05565	0,00215	-0,28234
Nerolidol	0,059	0,22201	0,15347
Hexanedioic acid, di-2-propenyl ester	0,09467	-0,16694	0,0466
1-Tridecanol	0,0285	0,06814	0,09943
n-Hexadecane	0,04542	-0,02697	0,104
9,12-Tetradecadien-1-ol	0,04455	0,00457	-0,24344
Hexadecanoic acid	0,0406	-0,24324	0,13372

Supplementary Table S5 Correlations between volatiles from differentially infested rice and factors identified after Multi-Factorial Analysis (MFA). Bolded values represent strong correlation between the chemical and the factor.
LRI=Linear Retention Index.

VOC emissions	LRI	Chemical	Factor 1	Factor 2	Factor 3
Exclusive Intact Rice	1024	2,2,8-Trimethyldecane	**-0.959473**	-0.25722	-0.098233
	1054	2-Methyldecane	**-0.959735**	-0.256163	-0.098016
	1101	Undecane	**-0.959621**	-0.256989	-0.098557
	1212	4,6-Dimethyldodecane	**-0.959944**	-0.255866	-0.098778
	1258	Linalool acetate	**-0.959042**	-0.257779	-0.098485
	1367	2-Butyl-1-decene	**-0.959657**	-0.256808	-0.098648
	1371	3-Methyltridecane	**-0.959978**	-0.256143	-0.098465
SZ-infested Rice	907	Heptanal	**0.966175**	0.229908	0.092283
	956	(E)-2-Heptanal	**0.971381**	0.219247	0.070127
	985	Hexanoic acid	**0.972285**	0.208076	0.093401
	1003	Octanal	**0.96971**	0.225882	0.081189
	1038	3-Octen-2-one	**0.971299**	0.215299	0.081184
	1074	1-Octanol	**0.977874**	0.191856	0.066667
	1098	2,5-Dimethylcyclohexanol	**0.969493**	0.226034	0.081422
	1179	Octanoic acid	**0.656975**	0.372427	0.12476
	1214	(2E,4E)-2,4-Nonadienal	**0.968581**	0.230564	0.080839
	1262	(E)-2-Decenal	**0.970133**	0.225094	0.074976
	1274	6-Undecanone	**0.986358**	0.154911	0.03636
	1280	2,6,11-Trimethyldodecane	**0.979461**	0.188682	0.028472
	1290	Pentyl hexanoate	**0.979855**	0.177247	0.072377
	1307	Undecanal	**0.979524**	0.186594	0.05698
	1320	(Z)-2-Tridecene	**0.967752**	0.227043	0.103011
	1326	Methyl decanoate	**0.98435**	0.166222	0.024773
	1336	Farnesane	**0.955132**	0.167297	0.236951
	1362	1-Undecanol	**0.96715**	0.205621	0.129535
	1375	2-Butyl-2-octenal	**0.970147**	0.219082	0.09617
	1480	(E)-2-Dodecen-1-ol	**0.979927**	0.137766	0.12512
	1492	1-Pentadecene	**0.925667**	0.120031	0.355579
Exclusive SZ-infested Rice	1969	Hexadecanoic acid	**0.56582**	-0.454792	-0.475476
Double-infested Rice	1090	1-Undecene	0.343689	**0.770289**	**0.529738**
	1124	2-Ethylhexanoic acid	0.342042	**0.78943**	**0.500028**
	1166	1-Nonanol	0.342902	**0.776696**	**0.521746**
	1259	γ-Octanolactone	0.341845	**0.790956**	**0.500062**
	1268	2-Butylcyclohexanone	0.342554	**0.788694**	**0.501412**
	1348	10-Methyl-trans-2-decalone	0.341682	**0.797402**	**0.587185**
Exclusive TC&SZ-infested Rice	931	α-pinene	0.240743	0.017177	**0.966553**
	1059	γ-terpinene	0.240791	0.017241	**0.96652**
	1287	(E)-Anethole	0.240826	0.017285	**0.966073**
	1306	(E)-2-Tridecene	0.24072	0.017622	**0.966017**
	1474	(Z)-8-Dodecen-1-ol	0.241035	0.016385	**0.965874**
	1538	Nerolidol	0.240799	0.017222	**0.966303**

VOC emissions	LRI	Chemical	Factor 1	Factor 2	Factor 3
Absent TC&SZ-infested Rice	1058	2-Octenal	**0.624754**	0.203879	**-0.750899**
	1174	Isomenthol	**0.654015**	0.062079	**-0.750612**
Exclusive CF&SZ-infested Rice	1257	1,3-Ditert-butylbenzene	0.153792	**0.899767**	-0.388012
	1275	Nonanoic Acid	0.155203	**0.896717**	-0.38672
	1363	γ-Nonanolide	0.15492	**0.897552**	-0.387106
	1419	Acetylenic Glycol	0.129477	**0.868815**	-0.378994
	1530	Tridecanal	0.154632	**0.898158**	-0.387337
	1642	9,12-Tetradecadien-1-ol	0.109896	**0.77175**	-0.3292
Common	994	1-Octen-3-ol	**0.976249**	-0.102285	0.044886
	1029	Limonene	-0.084059	-0.27496	**0.925175**
	1106	Nonanal	**0.984515**	0.120367	-0.031366
	1193	(E)-3-Dodecene	**0.767309**	-0.406833	-0.143936
	1200	Dodecane	**-0.894282**	-0.418786	0.001039
	1206	Decanal	**0.984168**	-0.117923	0.027448
	1242	1-Decanol	**0.687008**	**-0.608336**	-0.173179
	1295	1-Tridecene	**0.711357**	0.27533	0.249262
	1300	Tridecane	**-0.825459**	**-0.540425**	-0.145574
	1341	n-Heptylcyclohexane	**0.995292**	0.049156	-0.029897
	1354	5-Methyltridecane	0.01104	-0.447183	**-0.87863**
	1392	1-Tetradecene	**-0.514965**	**-0.776621**	-0.210658
	1399	Tetradecane	-0.459964	-0.795336	-0.228853
	1403	Dodecanal	**-0.683743**	0.288325	0.252993
	1447	1-Dodecanol	-0.126392	**-0.8610**08	0.088499
	1454	Geranylacetone	-0.128576	-0.054596	0.316449
	1467	2,6-Di-tert-Butyl-1,4-Benzoquinone	**0.789942**	0.108824	0.486276
	1500	Pentadecane	**0.80985**	-0.19164	0.401963
	1541	Hexanedioic acid, di-2-propenyl ester	**0.7639**	-0.193363	-0.332285
	1592	1-Tridecanol	0.170227	-0.12022	0.389939
	1599	Hexadecane	0.367305	-0.2413	0.12926
